# Integration of Transcriptome, miRNA-Omics, and Hormone Metabolism Analysis Reveals the Regulatory Network of *Camellia drupifera* Fruit Maturation

**DOI:** 10.3390/plants14213282

**Published:** 2025-10-27

**Authors:** Jin Zhao, Xue Sun, Yanqiang Yao, Ya Liu, Dongmei Yang, Huageng Yang, Jing Yu, Daojun Zheng, Yougen Wu

**Affiliations:** 1 School of Breeding and Multiplication (Sanya Institute of Breeding and Multiplication), School of Tropical Agriculture and Forestry, Hainan University, Sanya 572025, China; 18463652125@163.com (J.Z.);; 2 The Key Laboratory of Tropic Special Economic Plant Innovation and Utilization, Institute of Tropical Horticulture Research, Hainan Academy of Agricultural Sciences, Haikou 571100, China

**Keywords:** *Camellia drupifera*, fruit maturation, plant hormones, mRNA, microRNA

## Abstract

*Camellia drupifera* is an important woody oil crop with high economic and medicinal value. Fruit maturation is a complex process regulated by hormones and gene networks, yet its molecular basis remains unclear. Here, we integrated hormone profiling (IAA, GA_3_, ABA), transcriptomics, and miRNA-omics across three key stages: nutrient synthesis (S1), lipid accumulation (S4), and maturation (S7). During early development (S1), IAA and GA_3_ levels peaked, accompanied by the upregulation of growth-related genes (*AUX1*, *ARF*, *GID1*), which promote fruit growth. By maturation (S7), ABA content increased markedly, activating *PYR/PYL*, *PP2C*, and *ABF*, while IAA and GA_3_ declined. Transcriptome analysis revealed 45 key differentially expressed genes correlated with hormone levels. In parallel, miRNAs such as *miR393-z* (targeting *TIR1*) and *novel-m0146-5p* (targeting *ARF1*) were identified as regulators of hormone signaling and fruit maturation. Collectively, our results highlight a coordinated “hormone–miRNA–mRNA” regulatory network underlying *C. drupifera* fruit development. These findings provide new insights into the molecular regulation of fruit maturation and lipid accumulation in woody oil crops, offering a foundation for genetic improvement and efficient utilization of this species.

## 1. Introduction

*Camellia drupifera*, a plant belonging to the genus Camellia and the family Theaceae, is a significant woody oil crop [1,2]. This species is also commonly referred to as tea camellia, wild tea, or white-flowered tea. These plants are typically evergreen shrubs or small trees, distinguished by their seeds, which are rich in oil, giving them considerable economic value. The tea oil extracted from its fruit is laden with a variety of nutrients and possesses a unique, delicate fragrance. It stands as a premium, edible woody oil unique to China and has earned the moniker “Eastern Olive Oil” internationally [3]. Recognized alongside olive, palm, and coconut as a major economic crop, its ecological benefits, economic worth, and medicinal properties have garnered significant attention [4].

The development and maturation of fruit are processes regulated by the interplay of environmental factors and various plant hormones, which are crucial for both the quality and yield of the fruit. Endogenous hormones are involved in multiple aspects, including fruit growth, pigment formation, oil synthesis, and ripening-related softening [5,6]. Previous studies have shown that plant hormones play a key role in fruit development and maturation: indoleacetic acid (IAA) is involved in cell division and expansion; gibberellin (GA_3_) regulates fruit growth; and abscisic acid (ABA) promotes fruit ripening and abscission. Research has demonstrated that gibberellic acid 3 (GA3) and gibberellic acid 4 (GA4) can promote fruit enlargement [7,8,9,10,11]. Exogenous application of ABA has been shown to enhance anthocyanin accumulation in red-fleshed sweet cherry fruits and regulate the content of soluble sugars in the fruit [12]. In the pericarp and seeds of *C. drupifera*, the levels of four endogenous hormones (IAA, ZR, GA_3_, ABA) are relatively high during the early stages of fruit development. Still, the hormone contents in seeds and pericarp exhibit different trends thereafter, indicating that these four endogenous hormones play a crucial role in the development of young fruits [13,14]. Ethylene acts as a key signaling molecule during ripening, typically driving the process and the associated shifts in sugar metabolism and energy demand to promote the conversion of carbohydrates into oil and protein accumulations; cytokinins may, at different developmental stages, promote cell division and tissue expansion to provide the preconditioning for fruit development, thereby indirectly influencing metabolic reprogramming and resource allocation during ripening [15]. However, systematic studies on the dynamic changes in hormones during different stages of *C. drupifera* fruit development and the regulatory networks of downstream genes are still lacking.

Clarifying the expression patterns of hormone-related genes involved in fruit development is the primary step in revealing the hormonal molecular mechanisms underlying fruit ripening [16]. Previous studies have shown that IAA is the main factor regulating fruit development, while GA_3_ is involved in fruit development and enlargement. In non-climacteric fruits, ABA plays a crucial role during the ripening process [17,18,19]. Several IAA-regulated genes and regulatory signals have been identified, such as Auxin Response Factor 6 (*ARF6*) and Auxin Response Factor 8 (*ARF8*) [20]. In immature peach fruits, high concentrations of IAA and low concentrations of ABA synergistically promote fruit ripening, and key genes regulating IAA and ABA metabolism have been identified, including the ABA synthesis genes *PpNCED3* and *PpAAO3*, as well as the auxin transport and signaling genes *PpPIN* and *PpTIR1* [21]. GA_3_, by reshaping hormonal balance and activating the *VvRAX2*-mediated transcriptional regulatory network, collaborates with miRNA-lncRNA to regulate the cascade of genes, such as *GID2*, *SAUR*, and *ACS*, ultimately promoting cell expansion and volume increase in grape fruits [22]. The ripening of strawberry (a non-climacteric fruit) is primarily driven by ABA accumulation, a process co-regulated by the upregulation of *FaNCED1* and *FaABA2*, and the downregulation of *FaUGT75C1* and *FaCYP707A1*, which is closely associated with the expression of *FaMYB10* [23].

In recent years, the role of epigenetic regulation in fruit ripening has garnered increasing attention [24]. MicroRNAs (miRNAs), a class of non-coding RNAs approximately 21–24 nucleotides in length, precisely regulate gene expression at the post-transcriptional level by targeting mRNA for cleavage or inhibiting translation [25]. During the bud dormancy transition in apple, the *miR159-MYB* regulatory module influences the dormancy process through ABA signaling homeostasis, while miRNAs such as *miR858* participate in the regulation of the phenylpropanoid metabolic pathway [26]. In grape root development, differentially expressed miRNAs and their target genes regulate the signaling homeostasis of ABA and auxin through negative control, thereby modulating root architecture changes under root restriction (RR) conditions [27]. Notably, miRNAs can achieve cross-level regulation through a “hormone-miRNA-target gene” ternary interaction network. For instance, under drought stress in tomato plants, the expression of *miR159* is downregulated, specifically activating the *SlMYB33* transcription factor, which promotes the accumulation of osmoprotective compounds such as proline and putrescine, thereby enhancing drought resistance [28]. Sweet potato tuber development is regulated by a complex miRNA-mediated network, in which the auxin signaling pathway (via *miR319-TCP4*, *miR172-AP2*, etc.) serves as a key hub for inducing tuber initiation and expansion [29]. However, in *C. drupifera*, the molecular mechanisms underlying the synergistic regulation of lipid synthesis by hormones and miRNAs remain largely unexplored.

Hormones play a crucial role in the development of *C. drupifera* fruit and the accumulation of oil. However, the molecular network through which IAA, GA_3_, and ABA synergistically regulate fruit maturation remains unclear. Identifying hormone-responsive miRNA-mRNA regulatory modules can help elucidate the mechanisms by which hormones dynamically influence oil synthesis at different developmental stages. Therefore, this study combined with endogenous hormone determination, transcriptomics, and miRNA omics technology, systematically analyzed the hormone changes and gene expression profiles of the three key stages of fruit development (nutritional synthesis stage S1, fat accumulation stage S4, and maturity stage S7), and revealed the regulatory network between hormone signals and gene regulation during fruit development. This research offers new insights into the regulatory mechanisms of fruit maturation in woody oil crops, serving as a valuable reference for mechanistic studies of other woody oil crops.

## 2. Results

### 2.1. Analysis of Hormone Levels at Different Periods During the Ripening Process

**Figure 1:**
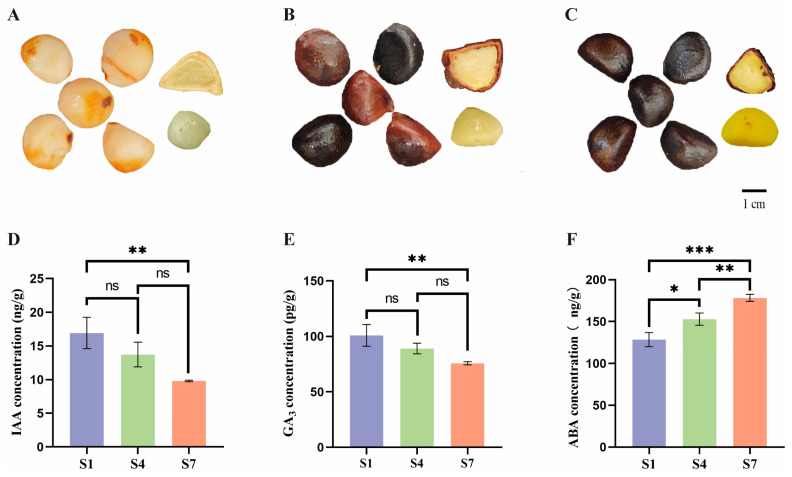
(**A**) Phenotype of *C. drupifera* seed kernel at S1 stage. (**B**) Phenotype of *C. drupifera* seed kernel at S4 stage. (**C**) Phenotype of *C. drupifera* seed kernel at S7 stage. (**D**) IAA content in *C. drupifera* fruit. (**E**) GA_3_ content in *C. drupifera* fruit. (**F**) ABA content in *C. drupifera* fruit. Blue represents the S1 stage, green represents the S4 stage, and pink represents the S7 stage. (*** *p* < 0.001, ** *p* < 0.01, * *p* < 0.05, ns > 0.05).

This study collected *C. drupifera* fruits at three different developmental stages and determined the endogenous hormone contents in the seeds at various periods, as shown in Figure 1. Phenotypic observations revealed that as the *C. drupifera* fruits developed, the seed coat color changed from white to brown, and the kernel color gradually transitioned from white to yellow. The distribution of endogenous hormones varied significantly across the three stages. At the S1 stage, the IAA content was the highest, reaching 16.94 ng/g, whereas at the S7 stage, it was 9.82 ng/g. The GA_3_ content in the fruit was highest at the S1 stage (100.90 pg/g), followed by the S4 stage, and lowest at the S7 stage (75.76 pg/g). The ABA content was highest in the fruit at the S7 stage, followed by the S4 stage, with the lowest content observed at the S1 stage.

### 2.2. Comparative Analysis of Genes Related to Plant Hormone Signal Transduction

**Figure 2:**
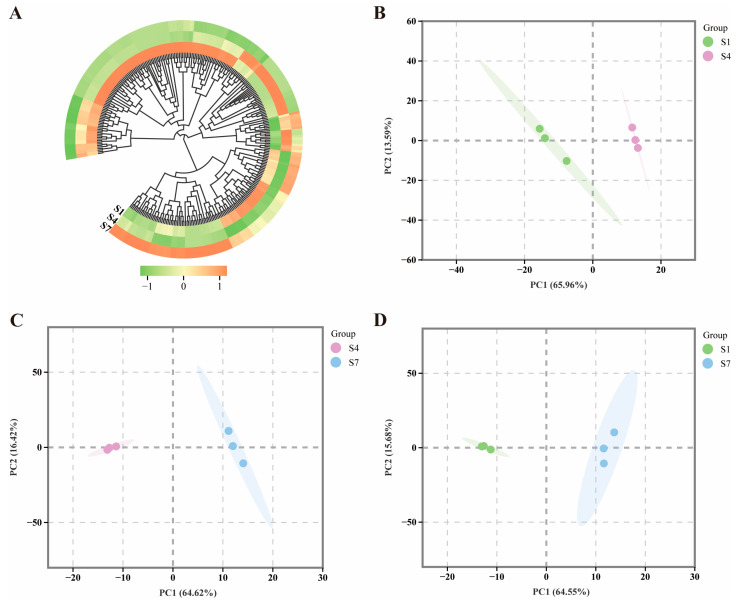
Analysis of Genes Related to Plant Hormone Signal Transduction in *C. drupifera* Fruits. (**A**) Clustering heatmap analysis of genes related to plant hormone signal transduction in *C. drupifera* fruits. Orange blocks represent upregulated genes, green blocks represent downregulated genes, and white blocks represent differentially expressed genes with average relative expression intensity. (**B**) PCA of S1 and S4 genes. (**C**) PCA of S4 and S7 genes. (**D**) PCA of S1 and S7 genes. Green represents the S1 group, purple represents the S4 group, and blue represents the S7 group.

The regulation of gene expression levels within the plant hormone signaling and transcription factor networks was assessed for the hormone-responsive gene set identified from RNA-seq data. To better understand the synthesis process of plant hormones, we utilized KEGG-enriched plant hormone signal transduction-related genes to analyze the expression patterns and differential expression of these genes (Figure S1). Based on the RNA-seq analysis of transcriptomes from three distinct periods, transcriptome data were retrieved from the NCBI Sequence Read Archive (SRA) under the BioProject accession number PRJNA1218904. A total of 298 genes associated with plant hormone signal transduction were identified across the three stages (S1, S4, and S7) (Table S1). A clustering heatmap (Figure 2A) illustrates the variations among these three stages. During the transition from the vegetative synthesis stage (S1) to the mature stage (S7), we observed a significant down-regulation of key auxin signaling genes, including the auxin receptor *TIR1* (Unigene0019971) and Auxin Response Factor *ARF1* (Unigene0052241). Conversely, key genes in the ABA signaling pathway, such as the transcription factor *ABF* (Unigene0028841), were significantly up-regulated in stage S7. The antagonistic expression patterns between auxin- and abscisic acid-related genes strongly support the hormonal changes observed during fruit maturation. The results of the Principal Component Analysis (PCA) revealed that the first two principal components (PC1 and PC2) collectively explained 80% of the total variance, indicating significant distinctions among the three stages (S1, S4, and S7).

**Figure 3:**
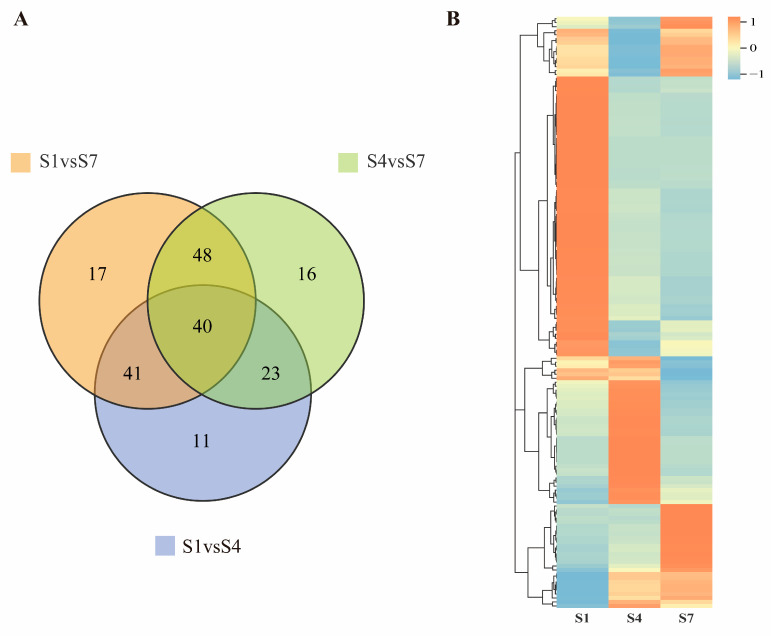
Analysis of differentially expressed genes related to plant hormone signal transduction in *C. drupifera* fruits. (**A**) Venn diagram analysis of the three comparison groups. (**B**) Heatmap analysis of differentially expressed genes (DEGs). Orange blocks represent upregulated genes. Blue blocks represent downregulated genes. White blocks represent differentially expressed genes with mean relative expression levels.

In the three comparison groups (S1 vs. S4, S4 vs. S7, and S1 vs. S7), 115, 127, and 146 differentially expressed genes (DEGs) were identified, respectively (Tables S2–S4). Venn diagram analysis results (Figure 3A) show 11 unique differentially expressed genes (DEGs) in the S1 versus S4 comparison, and a total of 44 unique DEGs across the three control groups. The heatmap (Figure 3B) indicates that more than 57% of DEGs exhibit the highest expression in the S1 group and the lowest expression in the S7 group. Expression of DEGs in the S4 group is lower than in S1 but higher than in S7.

### 2.3. Comparative Analysis of microRNAs Related to Plant Hormone Signal Transduction

**Figure 4:**
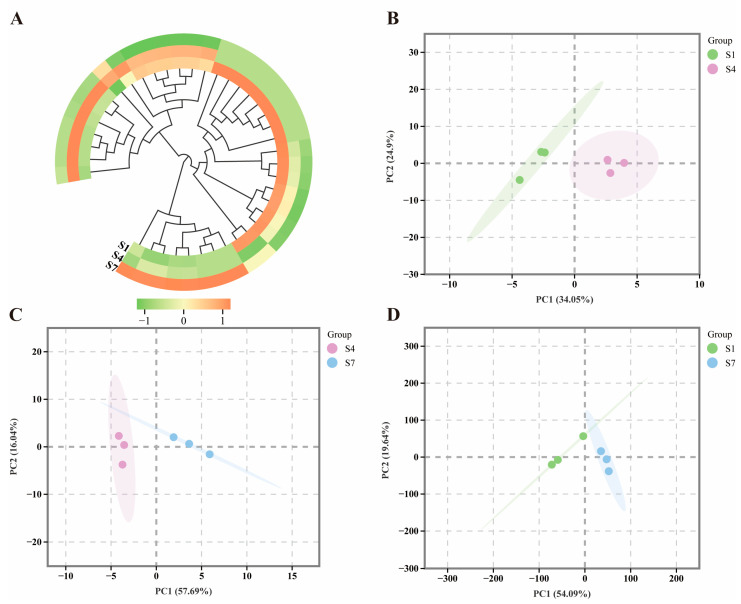
Analysis of microRNAs related to plant hormone signal transduction in *C. drupifera* fruits. (**A**) Clustering heatmap analysis of microRNAs associated with plant hormone signal transduction in *C. drupifera* fruits. Orange blocks represent upregulated genes, green blocks represent downregulated genes, and white blocks indicate differentially expressed genes with average relative expression intensity. (**B**) PCA of microRNAs in S1 and S4. (**C**) PCA of microRNAs in S4 and S7. (**D**) PCA of microRNAs in S1 and S7. Green represents the S1 group, purple represents the S4 group, and blue represents the S7 group.

A total of 40 microRNAs related to plant hormone signal transduction were detected across different periods (Table S5). The clustering heatmap results indicate differences in microRNAs regulating plant hormone signal transduction in *C. drupifera* fruit at various periods (Figure 4A). To better understand these differences in microRNAs involved in regulating plant hormone signal transduction in *C. drupifera* at different stages, PCA (Figure 4B–D) was performed. The PCA results reveal significant separation among the three distinct periods, with the two principal components (PCA1 and PCA2) accounting for approximately 60% of the total variance, indicating notable differences across the periods.

**Figure 5:**
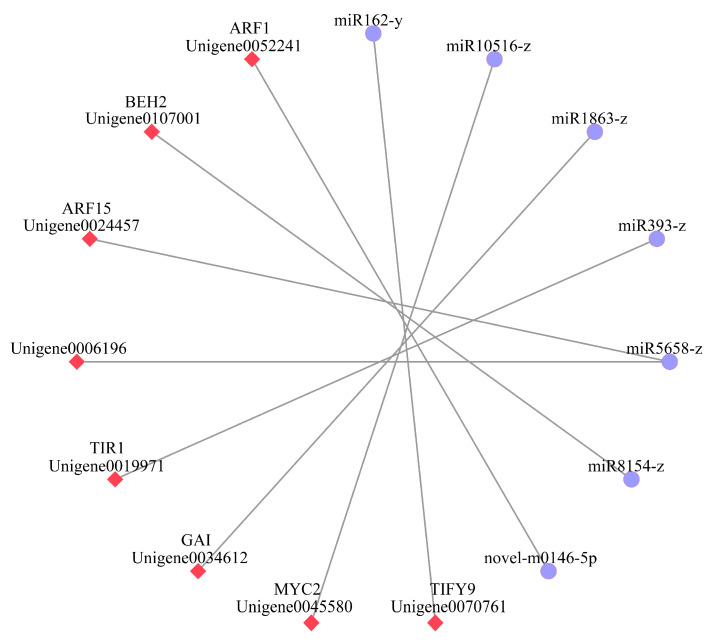
Network of differentially expressed miRNAs and their target genes. Blue circles represent miRNAs, and red diamonds represent target genes.

MicroRNAs can bind to target genes to inhibit translation or accelerate degradation, thereby achieving negative regulation of target gene expression. This negative regulatory mechanism is the primary functional mode of microRNAs, serving as a crucial basis for their biological effects. In these negative regulatory microRNA-target gene modules, a total of 7 microRNA-target gene negative regulatory modules were identified (Figure 5). Among them, 6 miRNAs each target a single differentially expressed gene, while 1 miRNA targets multiple differentially expressed genes related to hormone signal transduction in *C. drupifera*. Specifically, *miR162-y* negatively regulates *TIFY9* (Unigene0070761), *miR10516-z* negatively regulates *MYC2* (Unigene0045580), *miR1863-z* negatively regulates *GAI* (Unigene0034612), *miR393-z* negatively regulates *TIR1* (Unigene0019971), *miR8154-z* negatively regulates *BEH2* (Unigene0107001), *novel-m0146-5p* negatively regulates *ARF1* (Unigene0052241), and *miR5658-z* negatively regulates the expression of both (Unigene0006196) and *ARF15* (Unigene0024457).

### 2.4. Correlation Analysis of Differential Genes and Plant Hormone Content

**Figure 6:**
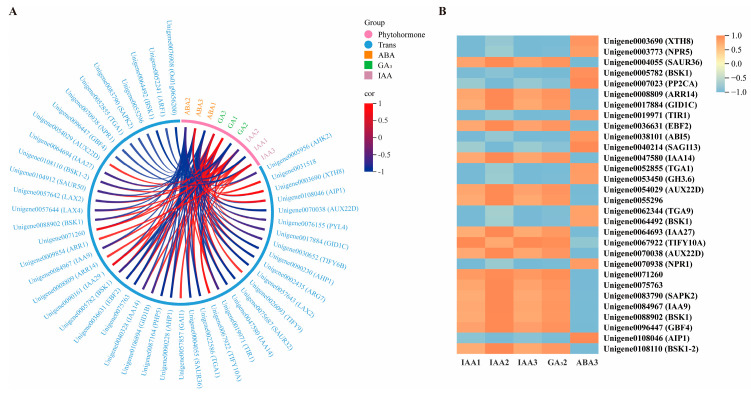
(**A**) Correlation analysis between differentially expressed genes in plant hormone signal transduction and plant hormone contents. Orange represents ABA, green represents GA_3_, purple represents IAA, blue represents differentially expressed genes, red lines indicate positive correlations, and dark blue lines indicate negative correlations. (**B**) Cluster thermogram analysis of genes related to hormone content and plant hormone signal transduction in fruits. Orange blocks represent up-regulated genes, blue blocks represent down-regulated genes, and white blocks represent the average relative expression intensity of differentially expressed genes.

To determine the regulatory roles of plant hormone signaling pathway–related DEGs on hormone levels, we conducted an integrative analysis of hormone signaling DEGs and endogenous hormone contents. Among 148 DEGs, 45 were identified as significantly correlated with the contents of IAA, GA3, or ABA. Among these, 16 DEGs showed a positive correlation with hormone content, 8 DEGs showed a negative correlation, and 21 DEGs exhibited both positive and negative correlations (Figure 6).

### 2.5. Analysis of Plant Hormone Signal Transduction Pathways in C. drupifera Fruit

**Figure 7:**
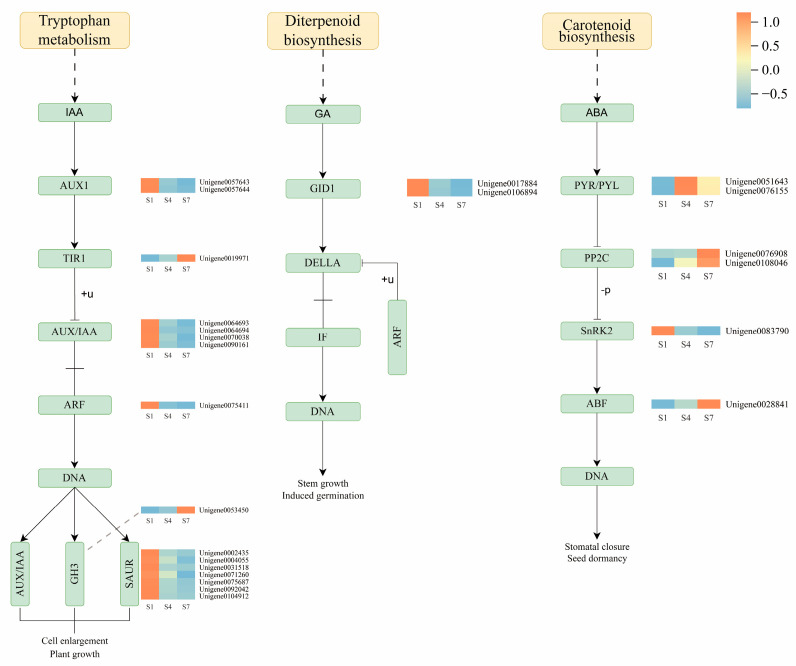
Synthesis and transduction of hormones in *C. drupifera* fruits at different stages. Orange blocks represent upregulated genes, blue blocks represent downregulated genes, and white blocks represent differentially expressed genes with average relative expression intensity. IAA: Indole-3-acetic acid; ABA: Abscisic acid; GA: Gibberellin.

In the IAA pathway (Figure 7), during the S1 stage, the upregulated genes involved in IAA synthesis include Unigene0057643, Unigene0057644 (*AUX1*), Unigene0064693, Unigene0064694, Unigene0070038, Unigene0090161, Unigene0040328 (*AUX/IAA*), Unigene0075411 (*ARF*), and Unigene0002435, Unigene0004055, Unigene0031518, Unigene0071260, Unigene0075687, Unigene0092042, Unigene0104912 (*SAUR*). Additionally, during the S7 stage, the IAA signal transduction genes Unigene0019971 (*TIR1*) and Unigene0053450 (*GH3*) were downregulated. In the GA pathway, the gibberellin receptor *GID1* (Unigene0017884 and Unigene0106894) was upregulated during the S1 stage (Figure 7). In the ABA pathway (Figure 7), the expression of ABA receptors *PYR/PYL* (Unigene0051643, Unigene0076155) was upregulated during the S4 stage. Three genes associated with protein phosphatase 2C (*PP2C*) and the *ABF* transcription factor family (*ABF*) (Unigene0076908, Unigene0108046, and Unigene0028841) were upregulated during the S7 stage. Conversely, Unigene0083790 (*SnRK2*) was downregulated during both the S4 and S7 stages.

The results indicate that in the IAA signaling pathway, multiple genes involved in IAA synthesis and signal transduction were upregulated during the S1 stage, including the auxin influx carrier *AUX1*, *AUX/IAA* family genes, auxin response factor *ARF*, and *SAUR* family genes. However, during the S7 stage, the expression of the IAA signaling pathway receptor gene *TIR1* and the auxin response gene *GH3* was downregulated. In the GA pathway, the gibberellin receptor gene *GID1* was upregulated during the S1 stage. In the ABA pathway, the ABA receptor genes *PYR/PYL* were upregulated during the S4 stage; meanwhile, three genes associated with the negative regulator of ABA signaling, *PP2C*, and the transcription factor *ABF* (Unigene0076908, Unigene0108046, Unigene0028841) were upregulated during the S7 stage. In contrast, the *SnRK2* kinase gene (Unigene0083790) was downregulated during both the S4 and S7 stages.

## 3. Discussion

### 3.1. Changes in the Hormone Content

Hormones play a critical regulatory role in the development of *C. drupifera* fruit [30]. Previous studies have shown that high levels of IAA in the early stage of fruit development promote cell division and contribute to the growth of young fruit. Meanwhile, GA_3_ content is relatively high during the early and middle stages of development, particularly in seeds, indicating that GA_3_ is closely associated with cell division and cell expansion during the middle stage [31,32,33]. In this study, the dynamics of endogenous hormones were measured across three developmental stages: S1 (nutrient synthesis stage), S4 (lipid accumulation stage), and S7 (maturation stage). The results revealed that the contents of IAA and GA_3_ peaked at the S1 stage and subsequently declined continuously, reaching their lowest levels at the S7 maturation stage. In contrast, ABA content increased steadily from its lowest level at the S1 stage, reaching a peak at the S7 stage. This changing trend is closely correlated with the fruit development process: high levels of IAA and GA_3_ at the S1 stage likely drive cell division and expansion by activating genes such as *AUX1* and *ARF* (Figure 7), thereby facilitating the morphological establishment of young fruit. From the S4 to S7 stages, the continuous accumulation of ABA coincided with the upregulation of genes such as *PYR/PYL* receptors, *PP2C*, and ABF (Figure 7), suggesting that ABA accumulation is associated with lipid accumulation and fruit maturation. Notably, the ABA peak at the S7 stage contrasted sharply with the low levels of IAA and GA_3_, which aligns with the hormone balance regulatory mechanism observed in non-climacteric fruit maturation.

### 3.2. Changes in Oil Content and Hormone Content

The oil content of fruits is dynamically regulated by a hormone interaction network [34]. Studies have shown that hormones dynamically modulate the oil content in oil palm mesocarp. Treatments with ABA and ethylene significantly upregulate the expression of the key transcription factor *EgWRI1-1* and its activator, thereby promoting the transcription of lipid synthesis genes (such as *EgDGAT2* and *EgFATB*) and increasing oil content. In contrast, IAA restricts lipid accumulation by enhancing the expression of the inhibitory factor *EgWRKY40*. Therefore, ABA and ethylene promote lipid accumulation through positive regulation of transcriptional cascades. At the same time, high levels of IAA and GA_3_ in the early stages of fruit development prevent premature initiation of oil storage processes by activating inhibitory factors [35]. Previous studies, through physicochemical analyses at different developmental stages (S1, S4, and S7), revealed significant changes. Oil content increased markedly, reaching 351.1% of the S1 level at S4 and peaking at 467.5% at S7, indicating a substantial accumulation of oil as the fruit matures [36]. We observed that in the early developmental stage (S1), high levels of IAA and GA_3_ activated the expression of genes such as *AUX1*, *ARF*, and *GID1* (Figure 7), primarily driving cell division and expansion, laying the morphological foundation for subsequent lipid synthesis. In the middle to late stages (S4 to S7), ABA content continued to rise, peaking at S7, while key genes in its signaling pathway (such as *PYR/PYL*, *PP2C*, and *ABF*) were also significantly upregulated (Figure 7). These changes synergistically promoted the expression of key enzymes involved in lipid synthesis, driving a rapid increase in oil content. At the final stage of maturation (S7), the peak ABA level contrasted sharply with the significant decline in IAA and GA_3_. This shift from growth hormones (IAA/GA) to maturation hormones (ABA) aligns with the maturation characteristics of non-climacteric fruits, suggesting that ABA may dominate the final processes of lipid conversion and accumulation during maturation by suppressing growth signaling pathways.

### 3.3. Combined Analysis of mRNA and microRNA in the IAA Pathway

The IAA signaling plays a central role in regulating fruit development and maturation in plants, and its dynamic balance is tightly controlled at multiple levels [37]. Previous studies have shown that in peanuts, the expression of numerous *AUX/IAA* genes corresponds to the dynamic changes in IAA levels during seed development [38]. In pomelo, a complex network composed of miRNAs (such as *miRn64* targeting *IAA9*) and mRNAs collaboratively regulates the fruit maturation process [39]. In tomatoes, the antagonistic interaction between *SlARF7/SlIAA9* and *SlDELLA* jointly controls fruit growth [40]. Additionally, in maize, nitrogen fertilization has been found to influence grain development by regulating key genes and novel miRNAs in the IAA biosynthesis and signaling pathways [41]. These studies collectively reveal the critical role of the IAA signaling pathway in controlling fruit development and the complexity of its regulatory network. In this study, we found that the content of IAA was the highest in the early stage of fruit development (S1 stage, nutritional synthesis stage), which corresponded well with the significant upregulation of multiple IAA biosynthesis and signaling genes (e.g., *AUX1*, *AUX/IAA*, *ARF*, *SAUR*) detected via transcriptome analysis (Figure 7). High levels of IAA promote the degradation of *AUX/IAA* proteins, thereby releasing *ARF* transcription factors to activate the expression of downstream growth-related genes. However, as the fruit enters the maturation stage (S7), the IAA content drops to its lowest level, and the expression of its key receptor gene, *TIR1* (*AUX/IAA*), and response gene *GH3* is significantly downregulated (Figure 7). This indicates that the IAA signaling pathway is inhibited during this stage, aligning with the fruit maturation process. More importantly, our combined analysis revealed the critical role of miRNAs in this dynamic regulation. During the S7 stage, we identified that *miR393-z* acts as a negative regulator of the *TIR1* gene. Its upregulation corresponded to the downregulation of its target gene *TIR1*, suggesting that *miR393-z* may weaken auxin signaling by inhibiting the perception of IAA (Figure 5). Additionally, a novel miRNA (*novel-m0146-5p*) was predicted to regulate *ARF1* negatively (Figure 5), suggesting that it may finely control the intensity of IAA responses during the S1 stage by modulating the level of *ARF* transcription factors. These findings demonstrate that miRNAs, such as *miR393-z* and *novel-m0146-5p*, collaboratively participate in the dynamic regulation of auxin signaling components (*TIR1*, *ARF*) during the transition of Camellia fruit from growth to maturation. This regulation has important potential implications for cell expansion, material accumulation, and transformation during fruit development.

### 3.4. Combined Analysis of mRNA and microRNA in Other Hormone Pathways

The functional antagonism between the plant hormones ABA and GA_3_ is manifested at two levels: metabolic regulation and signal interaction. At the metabolic level, environmental factors (such as light and temperature) induce transcription factors (e.g., *PIL5*, *ABI4*) that inversely regulate the expression of key enzyme genes, promoting ABA biosynthesis (*NCED*) and GA inactivation (*GA2ox*), while inhibiting ABA degradation (*CYP707A*) and GA biosynthesis (*GA3ox*) [42,43,44]. At the signaling level, core components directly interact; for instance, the SUMO ligase *SIZ1* simultaneously affects GA signaling component *SLY1* and ABA signaling component *ABI5*, while the NF-YC protein mediates the formation of a regulatory complex between the GA signaling repressor *DELLA* and *ABI5* [45,46]. ABA promotes its biosynthesis by upregulating the expression of *NCED1/3* genes and collaborates with ethylene signaling (e.g., *ETR2*, *PYR1* receptor genes) to regulate fruit maturation processes. Additionally, microRNA-mediated post-transcriptional regulation influences ABA-driven anthocyanin accumulation [47]. In this study, a clear ABA/GA_3_ antagonistic pattern was also observed in Camellia fruits. Specifically, from S4 to S7, the ABA content increased continuously, reaching a peak at S7 (Figure 1F). This trend aligns with ABA’s role as a maturation-promoting factor, and its accumulation likely drives fruit maturation and lipid transformation by activating downstream signaling elements, such as the significantly upregulated *PP2C* (Unigene0076908, Unigene0108046) and *ABF* transcription factor (Unigene0028841) at S7 (Figure 7). In contrast, the content of GA_3_ S1 was the highest, and then continued to decline significantly, reaching the lowest level at S7 (Figure 1E). The high level of GA_3_ at S1 corresponds to the elevated expression of the gibberellin receptor gene *GID1* (Unigene0017884, Unigene0106894) (Figure 7), suggesting that GA_3_ signaling dominates growth phase processes such as cell division and expansion during early fruit development. Its subsequent decline may create conditions for ABA-mediated maturation processes. In summary, the increase in ABA content and the decrease in GA_3_ content form a dynamic antagonistic relationship, with the two hormones jointly and precisely coordinating the critical developmental transition of Camellia fruits from growth phase to maturation and lipid accumulation. The analysis in this study is mainly correlational. Confirming the causal relationship of key regulatory pathways, such as the one involving *miR393-z* and *TIR1*, needs further validation through functional experiments like gene editing, overexpression, or silencing. The regulatory network model is incomplete. This research only examined three hormones: IAA, GA_3_, and ABA. Future studies should include other hormones, such as ethylene and cytokinin, along with their signaling pathways, to develop a more comprehensive regulatory map.

## 4. Materials and Methods

### 4.1. Plant Material

In 2023, fresh fruits of *C. drupifera*’ Wanhai 3′ were collected from the *C. drupifera* plantation in Hongmao Town, Qiongzhong City, Hainan Province (19°0′56″ N, 109°42′59″ E). The region boasts a mild climate, characterized by an annual average temperature of 22–24 °C, a yearly sunshine duration of 1600–2000 h, and an annual precipitation range of 2200–2444 mm. Sampling was conducted from August to November 2023, and fruit development was divided into three stages: the nutrient synthesis stage (S1, 270 days after pollination), the lipid accumulation stage (S4, 315 days after pollination), and the maturity stage (S7, 360 days after pollination) [36,48]. Fruits were collected from the periphery of the canopy of nine selected trees, with three biological replicates each comprising fruits from three trees, chosen to minimize microenvironmental variation and to represent uniform developmental stages. For each developmental stage, the collected fruits were peeled, and the seeds were divided into two portions. One portion was peeled to remove the seed coat, immediately wrapped in aluminum foil, rapidly frozen in liquid nitrogen, and subsequently stored at −80 °C for further analysis.

### 4.2. Determination of Endogenous Hormones in C. drupifera Fruits at Different Stages

For hormone extraction, weigh about 2 g of seed kernel and place it in a 10 mL centrifuge tube. Subsequently, 5 mL of precooled PBS buffer (pH 7.2–7.4) was added, and the samples were stored overnight in the dark at 4 °C for extraction. Centrifuge at 10,000 rpm/min for 10 min at 4 °C and use the supernatant as the test solution. Fruits were determined by enzyme-linked immunosorbent assay (ELISA). The IAA, GA3, and ABA in the sample were, respectively, combined with the micropores coated with anti-IAA, GA3, and ABA. The HRP-labeled detection antibody was then combined with the substrate to develop color. Finally, the OD value was determined at 450 nm by an enzyme-labeled instrument, and the concentration was calculated by a standard curve. Samples were properly extracted and diluted, and standard curves were established for different batch operations to ensure accuracy. The standard curve of IAA is y = 8.48 2 − 5.1088 x + 1.978, R^2^ = 0.9944; the standard curve of GA3 is y = 7.2797 x^2^ + 33.826x-3.6167, R^2^ = 0.9997; and the standard curve of ABA is y = 14.886 x^2^ − 3.5127x + 2.7975, R^2^ = 0.9946. The reagent kit used was an ELISA kit (Shanghai Yuanju Biotechnology Centre, Shanghai, China).

### 4.3. RNA Extraction, Library Construction, and Sequencing

Total RNA was extracted from samples using the Trizol reagent kit (Thermo Fisher Scientific, Waltham, MA, USA) according to the manufacturer’s instructions. The quality and integrity of the isolated RNA were assessed with an Agilent 2100 Bioanalyzer (Agilent Technologies, Santa Clara, CA, USA) and by agarose gel electrophoresis. Messenger RNA (mRNA) was enriched from total RNA using oligo(dT) magnetic beads, and ribosomal RNA (rRNA) was removed using the Ribo-Zero kit (Illumina, San Diego, CA, USA). The enriched mRNA was fragmented, and first-strand cDNA was synthesized by reverse transcription, followed by second-strand synthesis with DNA polymerase I. Purification of the double-stranded cDNA and ligation of Illumina sequencing adapters were performed using the QIAquick purification kit (Qiagen, Hilden, Germany). The resulting libraries were validated by agarose gel electrophoresis and PCR, and subsequently sequenced on the Illumina NovaSeq 6000 (Illumina, San Diego, CA, USA) platform. For mRNA bioinformatics analysis, raw reads were quality-filtered using FASTQ (v0.18.0) to obtain clean reads. Clean reads were assembled de novo with Trinity, and differential expression analysis between the two groups was conducted using DESeq2 (version 1.36.0). Differentially expressed genes (DEGs) were defined as those with an absolute fold change of at least 2 and a false discovery rate (FDR) < 0.05. Functional annotation and pathway enrichment were performed using Gene Ontology (GO) and Kyoto Encyclopedia of Genes and Genomes (KEGG) databases, with enrichment highlighting DEGs involved predominantly in signaling transduction and metabolic pathways.

### 4.4. Construction and Sequencing of miRNA Libraries

MicroRNA (miRNA) libraries were prepared from total RNA by first enriching RNA species in the 18–30 nucleotide range via polyacrylamide gel electrophoresis (PAGE), followed by ligation of 3′adapters and enrichment of 36–44 nucleotide fragments; subsequently, the 5′adapters were ligated. cDNA libraries were constructed and size-selected to 140–160 base pairs by PCR amplification, and the final products were sequenced on the Illumina NovaSeq 6000 platform. Reverse transcription–PCR amplification was used to amplify the adapters. For miRNA annotation and expression analysis, raw reads were processed with FASTQ (v0.18.0) to generate clean reads. To remove RNA species other than miRNA, reads were filtered by aligning to GenBank (v209.0) and Rfam (v11.0) to exclude rRNA, scRNA, snoRNA, tRNA, and snRNA. Known miRNAs were identified by alignment to miRBase (v22), and novel miRNAs were predicted with miRDeep2 based on genomic location and hairpin structure. Expression levels were quantified as transcripts per million (TPM) using the formula TPM = (miRNA counts/total clean reads) × 10^6^. Prior to differential expression analysis, miRNAs with less than 1 TPM in at least three samples were filtered out. Differential expression analysis was performed with edgeR, with miRNAs exhibiting a |log2(Fold Change)| ≥ 1 and an adjusted *p*-value (False Discovery Rate, FDR) < 0.05 considered differentially expressed. Predicted targets of miRNAs were identified using patmatch (v1.2), complemented by sequence feature and family information from TargetScan. Target genes were subjected to GO and KEGG annotations. miRNA target networks were constructed by integrating miRNA and target gene differential expression and interaction data; Pearson correlation coefficients were calculated for miRNA–target pairs using expression data from all nine samples (three biological replicates across three developmental stages). Pairs with Pearson correlation coefficient (r) < −0.7 and an adjusted *p*-value (False Discovery Rate, FDR) < 0.05 were considered to exhibit significant negative regulation and were visualized in Cytoscape (v3.6.0). Functional enrichment analyses were performed on the selected target genes to elucidate potential biological roles.

### 4.5. Statistical Analysis

Excel 2016 and IBM SPSS Statistics 26 software were used for data statistics and significant difference analysis. We used GraphPad Prism 8 software to draw the chart, and TBTools v2.056 software to make the heat map. Graphics and charts are processed using Adobe Illustrator 2024 and Adobe Photoshop 2024 software.

## 5. Conclusions

In summary, this study analyzed the dynamic regulation of endogenous hormones (IAA, GA_3_, ABA) in conjunction with miRNA and mRNA during the three developmental stages of *Camellia* fruits to explore maturation mechanisms. In the early growth stage (S1), high levels of IAA and GA_3_ promoted cell division and expansion by upregulating genes like *AUX1* and *ARF*. In contrast, during the maturation stage (S7), a significant increase in ABA content, along with upregulated genes such as *PYR/PYL* and *PP2C*, appeared to facilitate lipid accumulation and initiate maturation. Additionally, key miRNAs (e.g., miR393-z) were identified to fine-tune hormone signaling by negatively regulating core genes of the IAA pathway, such as *TIR1* and *ARF1*. A correlation analysis further identified 45 differentially expressed genes significantly associated with the hormone levels. In conclusion, this study unveils a “hormone-miRNA-mRNA” regulatory network in *Camellia* fruit maturation, offering important theoretical insights into the development of woody oil fruits.

## Data Availability

The data will be made available on request. The datasets presented in this study can be found in online repositories. The names of the repository/repositories and accession number(s) can be found at the following link: https://www.ncbi.nlm.nih.gov/bioproject/PRJNA1218904, accessed on 4 February 2025.

## Supplementary Materials

The following supporting information can be downloaded at: https://www.mdpi.com/article/10.3390/plants14213282/s1. Figure S1. Unique genes in the top 30 KEGG enrichment analysis. Table S1: Transcriptome data from three sets of samples; Table S2: S1 vs. S4 Differential Expression Analysis; Table S3: S4 vs. S7 Differential Expression Analysis; Table S4: S1 vs. S7 Differential Expression Analysis; Table S5: miRNA-seq data from three sets of samples.
