# Supplementary figures and images for: Integration of Transcriptome, miRNA-Omics, and Hormone Metabolism Analysis Reveals the Regulatory Network of Camellia drupifera Fruit Maturation
Source: Plants (Basel). 2025 Oct 27;14(21):3282. doi: 10.3390/plants14213282 (PMC12609256; doi:10.3390/plants14213282)

## Supplementary Figure

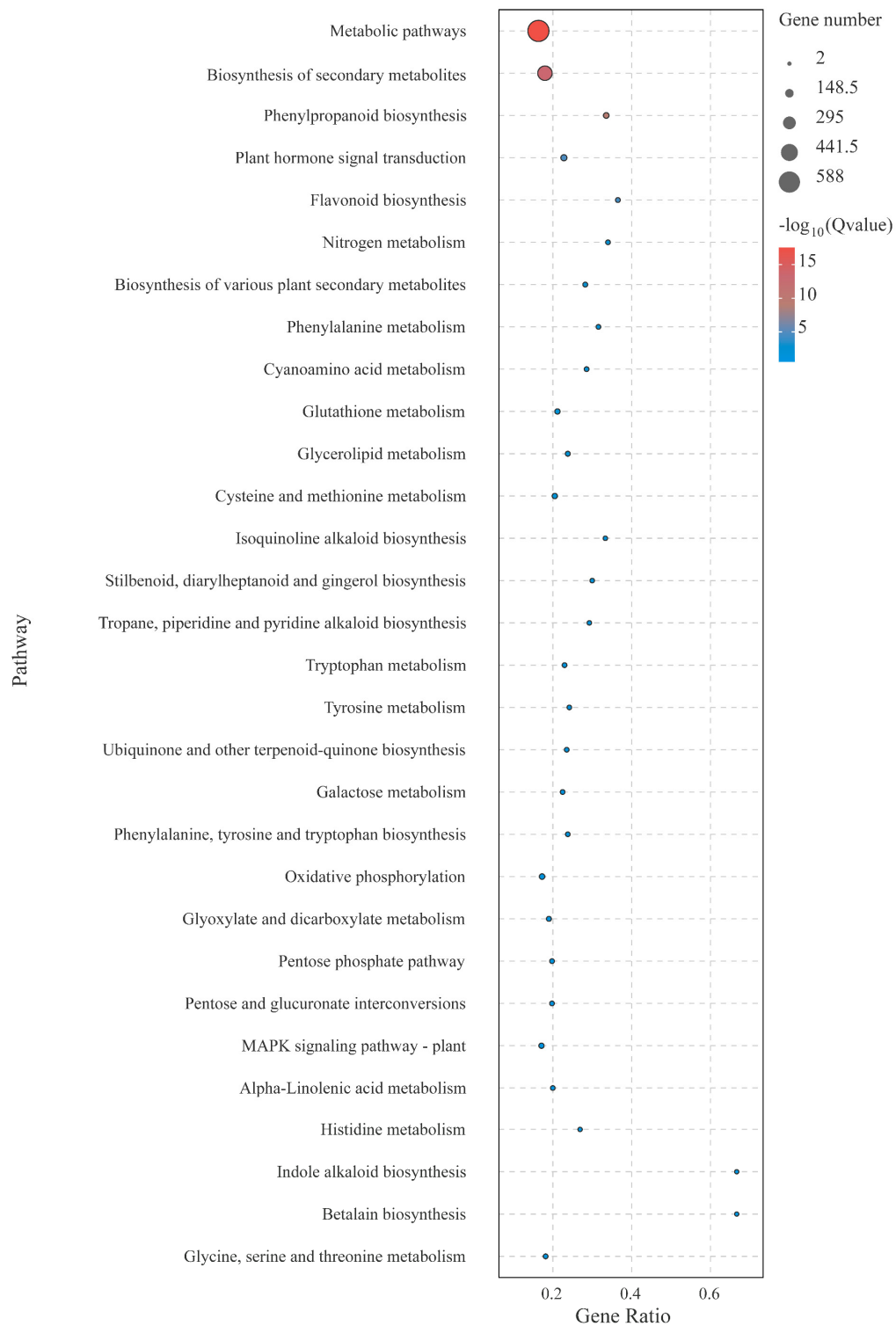

**Figure S1.** Unique genes in the top 30 KEGG enrichment analysis.

Supplement: Supplementary file 1 [file plants-14-03282-s001.zip › Supplementary Figure.pdf]
